# Rural health dialogue for the sustainability of help-seeking behaviors among older patients: grounded theory approach

**DOI:** 10.1186/s12877-023-04401-3

**Published:** 2023-10-18

**Authors:** Ryuichi Ohta, Chiaki Sano

**Affiliations:** 1 Community Care, Unnan City Hospital, 96-1 Iida, Daito-cho, Unnan, 699-1221 Japan; 2 Department of Community Medicine Management, Faculty of Medicine, Shimane University, 89-1 Enya cho, Izumo, Shimane Prefecture, 693-8501 Japan

**Keywords:** Community participation, Grounded theory approach, Help-seeking behavior, Healthcare sustainability, Rural health dialogue, Rural older patients

## Abstract

**Background:**

Help-seeking behaviors (HSBs) are essential for disease prevention and health promotion. Dialogues with peers and medical professionals can improve HSBs, both qualitatively and quantitatively. Rural communities lacking healthcare resources require effective HSBs for healthcare sustainability. The current study aimed to investigate the effect of health dialogues between medical professionals and rural citizens on their HSBs.

**Methods:**

All procedures complied with the Declaration of Helsinki and its subsequent amendments. The Unnan City Hospital Clinical Ethics Committee approved the study protocol (No. 20,220,002). A grounded theory approach was employed for the health dialogue participants in rural communities. Health dialogues with family physicians were conducted once a month at rural community centers. The dialogues and focus group interviews were recorded and coded to investigate changes in participants’ perceptions and behaviors regarding HSBs.

**Results:**

Twenty-one dialogues were conducted in two rural community centers, with a total of 112 participants. The average age of the participants was 70.2 years (standard deviation = 5.4), with 24% being males. Analysis of the grounded theory approach revealed four themes, namely joy-driven dialogue driving the realization of HSBs, reflection on personal HSBs through learning from others, revising HSBs based on rural social resources, and familiarity with physicians, hence motivating safe and secure HSBs.

**Conclusions:**

Mitigation of barriers between citizens and medical professionals and improvement of psychological safety in communities can drive effective HSBs in rural communities.

## Background

Help-seeking behaviors (HSBs) are essential for preventing diseases and promoting health conditions, and these are driven by social support and enhanced intentions toward HSBs [1–3]. HSBs are behaviors that address people’s symptoms and anxieties. When people approach symptoms and anxieties [4, 5], they are influenced by various social, demographic, and socioeconomic factors [6, 7]. Effective HSBs can mitigate difficulties in life and improve the overall quality of life (QOL) [8]. Social support, self-efficacy, and intention toward HSBs are essential [9] for overcoming such difficulties, leading to the effective use of healthcare resources [7, 10]. Dialogue between lay people and healthcare professionals is one of the social resources driving support and intention [11].

Dialogue with peers and medical professionals can improve HSBs’ quality and quantity, and it is particularly essential for older people. In communities, people engage in dialogue with others regarding their health conditions and may modify their HSBs based on others’ suggestions [12, 13]. In particular, older people change their HSBs based mainly on their relatives and family’s dialogues and suggestions owing to limited access to communication resources and interactions with others [9, 14]. In addition, older people frequently use medical facilities, where dialogues with medical professionals can modify their HSBs for common critical symptoms [15–17]. Effective advice from physicians can motivate older adults to act based on their symptoms [18, 19]. The dialogue between older people and medical professionals should be further driven in medical institutions and communities to improve HSBs [18].

In the rural context, the lack of healthcare resources necessitates effective HSBs and dialogues between older people and medical professionals. Older people in rural communities have limited access to medical institutions, and hence, outreach projects regarding HSBs are essential [18–20]. A previous review suggested that rural contexts need additional outreach projects for older people’s HSBs and collaborations among physicians and citizens for better HSBs [18, 21]. For effective healthcare in rural contexts, physicians need to reach out to communities, have dialogues with older people regarding HSBs, and improve their intention for HSBs, leading to the effective usage of healthcare resources and a better QOL [18].

For effective dialogue with rural citizens, the involvement of family physicians is vital. Family physicians specialized in family medicine systematically treat patients from biopsychosocial aspects [20, 22]. Dialogues with rural citizens regarding HSBs may demand knowledge from multiple medical fields and social sciences, such as sociology and public health [5, 22]. To date, the effects of health dialogues between older rural citizens and their family physicians on the citizens’ HSBs have not been investigated. In this study, we aimed to investigate how health dialogues regarding HSBs among family physicians and rural older people could affect their attitudes toward their symptoms. In other words, we investigated the effect of health dialogues between medical professionals and rural citizens on their HSBs.

## Materials and methods

This qualitative study used a grounded theory approach regarding the health dialogues of participants in rural communities. Choosing the constructivist aspect of the grounded theory informed our decisions and analysis by emphasizing the mutual creation of knowledge by the researcher and participants. For instance, the constructivist grounded theory focuses on the researcher’s reflections and interactions with the participants as a part of the data, which, in our study, helped in gaining deeper insights into the participants’ experiences and perspectives regarding HSBs. This theoretical perspective enabled us to develop a rich, context-based understanding of how dialogues influence HSBs in rural settings, acknowledging the diversity and complexity of participants’ experiences.

### Setting

The study was conducted in Unnan City, Shimane Prefecture, Japan. This city, situated in the eastern part of Shimane, borders Hiroshima Prefecture in the south and spans a total land area of 553.1 km^2^, accounting for 8.3% of Shimane Prefecture. The landscape of Unnan City is predominantly covered by forests, contributing to its characterization as a rural area, significantly distanced from urban centers.

A survey conducted in 2020 revealed that Unnan City had a total population of 36,007, with a notable 40.01% of the population being older than 65 years. This demographic trend, common in rural areas, reflects the city’s social organization and structure, which consists of 30 multifunctional autonomous communities. Each community is equipped with diverse functions to address various social issues and needs, reflecting demographic trends and social organization common in rural areas of Japan [23].

Politically, Unnan City operates under the local governance structure of Shimane Prefecture, with an emphasis on community participation and citizen empowerment. Local authorities manage public services, including healthcare, facilitated through different community organizations, underscoring the city’s distinctive political and social framework.

In assessing the healthcare landscape, Unnan City faces challenges typical of rural settings, such as limited access to healthcare facilities and professionals. Residents often rely on local clinics and healthcare centers, and they may need to travel to neighboring areas for specialized medical services. The availability of healthcare professionals is constrained, necessitating reliance on visiting practitioners for certain medical needs [23].

In characterizing the rurality of Unnan City, it is pivotal to consider the interplay between its traditional rural features and the influences of urbanization and digital technology. Despite its expansive forested areas and a significant proportion of the population engaged in agriculture and forestry, the city has experienced the penetration of digital technologies. This integration impacts the socio-economic dynamics and lifestyles of its inhabitants, as evidenced by the multifunctional autonomous communities utilizing digital platforms for managing social issues and facilitating communication.

The communities employ a blend of traditional protocols and modern digital solutions to manage functions such as community organization, healthcare, and continual education. Community organization, in particular, emphasizes citizen empowerment, effective utilization of local resources, and problem-solving through local leaders, often facilitated by digital communication tools [23].

By incorporating digital technologies, Unnan City exemplifies a hybrid rural–urban landscape. The traditional definition of ‘rural’ is nuanced by the presence of urban features and digital connectivity, shedding light on the evolving nature of rural spaces in the contemporary world. The intricate interplay between rural characteristics; urban influences; and the unique geographic, social, and political context of Unnan City provides a rich backdrop for our study and the health dialogues conducted within the communities.

### Participants

Between April 2022 and March 2023, 112 citizens from rural communities participated in health dialogues. Purposive sampling was performed to address the research purposes of ethnographic and semi-structured interviews. Two communities (Tane and Matsukasa) were chosen based on their substantial distance from the central part of Unnan City. The inclusion criteria were the residents of Tane and Matsukasa communities, age 18 years or older, ability to provide informed consent and willingness to participate in the study, and absence of fever and other COVID-19 symptoms at the time of the study. The exclusion criteria were non-residents of Tane and Matsukasa communities, individuals below 18 years of age, inability to provide informed consent or unwillingness to participate in the study, and presence of fever or other COVID-19 symptoms at the time of the study. The study participants from each community center were briefed about the study and provided informed consent to participate, adhering to the above criteria. The participants wore facemasks and maintained a distance of more than two meters between them.

### Rural health dialogue

A rural health dialogue among family physicians and older rural people was conducted to share experiences and knowledge regarding HSBs in rural communities. Health dialogues were held once a month in each community center. The number of participants in each session was between 10 and 12. Each session had one health theme, namely chronic diseases (hypertension, dyslipidemia, diabetes, and chronic obstructive pulmonary diseases), exercise, food, sleep, joint pain, constipation, polypharmacy, multimorbidity, and palliative care. The duration of each session was approximately 60 to 90 min.

In each health dialogue, family physicians initially explained one health topic regarding the prevalence of diseases in communities, related diseases, treatments, and concrete experiences regarding diseases. Next, participants voluntarily shared their experiences regarding health issues. Family physicians allowed the participants to share their experiences and answered prompted questions. Furthermore, based on the participants’ experiences, family physicians inquired about how they acted on their symptoms regarding HSBs. Various ideas and behaviors on how to control and approach the symptoms were shared; the family physicians respected each behavior and the reason underlying their HSBs. Finally, family physicians shared examples of effective behaviors to control symptoms and prevent exacerbations of symptoms and diseases.

### Measurements

#### Ethnographic and focus group interviews

Observational and focus group interviews, consistent with grounded theory methodology, were conducted with the participants. Observations were carried out to understand the context and interactions among community-center participants, informing the subsequent focus-group interviews. While our observational strategies were inspired by ethnographic methods, they were conducted with the purpose of informing our grounded-theory approach. These observations provided rich contextual data that aided in the development of our interview guide and contributed to the depth and relevance of our subsequent focus-group discussions. Only rural citizens were included in the ethnographic study. The specialties of the researcher included family medicine, medical education, rheumatology, and public health. Researchers worked in rural clinics, observed the interactions among community center participants, and took field notes during the study. One of the researchers, R.O., facilitated focus group interviews after each health dialogue in the community center room. The interview guide included the following four questions: What did you think of the health dialogue with medical doctors? How did you consider help-seeking behavior in your community? How did the health dialogue assist in changing your help-seeking behavior? How can you change your help-seeking behavior? Do you have any idea on how to improve the health dialogue? Each interview lasted approximately 60 min and was recorded and transcribed verbatim.

#### Analysis

The inductive grounded theory approach was used to investigate how participants in rural health dialogues changed their perceptions and behaviors regarding HSBs [24]. After reading the contents of the field notes and conducting in-depth semi-structured interviews, the first researcher coded the contents and developed codebooks based on repetitive readings of field notes, as the initial coding. The study used the process and concept coding method [24, 25]. Thus, the researcher induced, merged, deleted, and refined the concepts and themes by going back and forth between the research materials and initial coding for axial coding. Axial coding elaborates on the concepts and themes while refining the codes. For triangulation, the first and second researchers discussed the concepts and themes. The interview contents were analyzed iteratively during the research period for theoretical saturation. Finally, the theory was discussed by both researchers, who ultimately agreed on the final themes.

#### Ethical consideration

Participant anonymity and confidentiality were ensured throughout the study. All participants provided written informed consent before participating in the conferences and answering the questionnaires. All procedures complied with the Declaration of Helsinki and its subsequent amendments. The Unnan City Hospital Clinical Ethics Committee approved the study protocol (No. 20,220,002).

## Results

### Outcome of the grounded theory approach

On analyzing the themes and concepts emerging from the data, we identified the transformative impact of health dialogues on participants’ HSBs in rural communities as the central phenomenon of our study. This phenomenon encapsulates the evolution of individual attitudes and behaviors toward health, stemming from the joy-driven dialogues, reflection and realization of personal HSBs through learning from others, revising HSBs in the context of available rural social resources, and fostering a sense of familiarity with physicians, thereby improving motivation for safe and secure HSBs (Table 1).

**Fig. 1:**
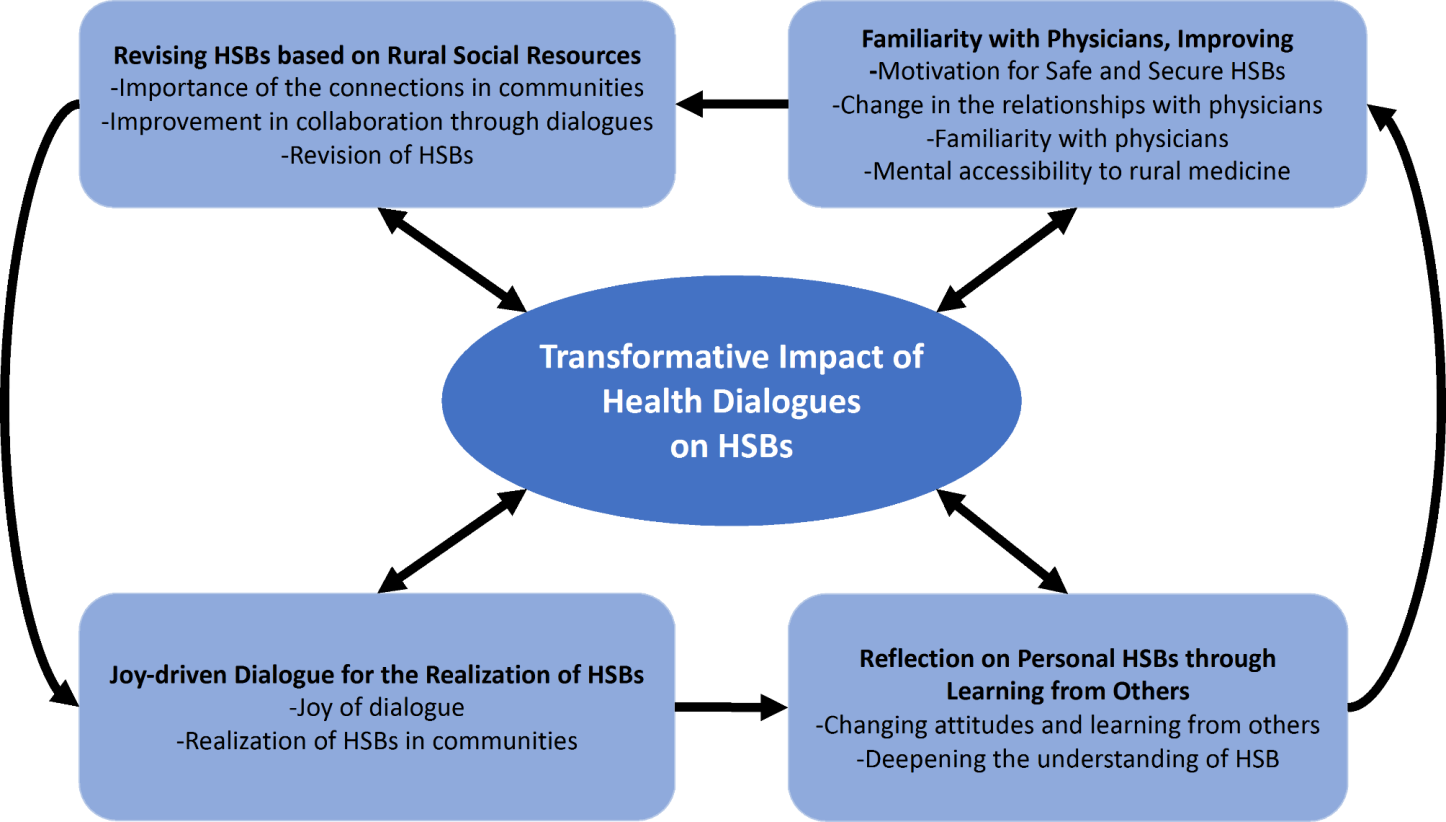
Conceptual depiction of the transformative impact of health dialogues as the central phenomenon, driving changes in perceptions and behaviors regarding HSBs in rural communities, and its interplay with the identified themes

Our findings surrounding this central phenomenon elucidate how health dialogues serve as a catalyst for participants to reflect on and reconsider their health behaviors, revealing discrepancies in attitudes and enabling the discovery of alternative health management strategies. This, in turn, contributes to a more collaborative and resourceful approach to health management in rural settings, highlighting the significance of community interactions and the critical role of physician relationships in influencing perceptions and access to healthcare. The central phenomenon is visually represented and integrated into Fig. 1, illustrating its foundational role in connecting the identified themes and shaping the overall narrative of HSB transformation in rural communities.

Figure 1. Conceptual depiction of the transformative impact of health dialogues as the central phenomenon, driving changes in perceptions and behaviors regarding HSBs in rural communities, and its interplay with the identified themes.

**Table 1:** Results of the grounded theory approach

Theme	Concept
Joy-driven dialogue toward the realization of HSBs	Joy of dialogue
	Realization of HSBs in communities
Reflection on personal HSBs through learning from others	Changing attitudes and learning from others
	Deepening the understanding of HSB
Familiarity with physicians, thereby improving motivation for safe and secure HSBs	Change in the relationships with physicians
	Familiarity with physicians
	Mental accessibility to rural medicine
Revising HSBs based on rural social resources	Importance of the connections in communities
	Improvement in collaboration through dialogues
	Revision of HSBs

### Joy-driven dialogue for the realization of HSBs

The health dialogue allowed participants to share their health experiences openly in the community. As they aged, they lost the opportunity to talk to each other; therefore, they felt joy during the dialogue. Participant 2 stated, “I enjoyed talking with other in different communities. I wanted to talk about health, but there were no opportunities. This opportunity for dialogue is essential for our health.” Previously, they felt that talking openly about health might invade their privacy. Through the joy of dialogue, they realized that sharing their healthcare difficulties was beneficial for their HSBs in communities. Participant 5 stated, “Joy can be important in this dialogue. The participants could share their difficulties in health. Initially, I considered that privacy was challenging in this dialogue. However, dialogue with joy about health allowed me to openly discuss my difficulty.”

Furthermore, the dialogue allowed the participants to understand others’ ideas and behaviors regarding their health problems, some of which were different or new among the participants. They could get the time required for realizing their specific behaviors regarding their health problems. Participant 13 stated, “I was surprised to listen to others’ behaviors regarding their health. They completely behaved differently from me. I considered that they should take rest, but they are healthy now. So, I realized that my health behaviors could be wrong or modified.”

### Reflection on own HSBs by learning from others

While health dialogues served as a catalyst for reflection on HSBs among participants, note that other social determinants of health, such as socioeconomic status, access to healthcare, and education, also play a crucial role in shaping these behaviors. The ones that had a dialogue regarding HSBs in communities before participating in this dialogue could understand other citizens’ behaviors using multiple resources. Participant 4 stated, “Some of the participants did know new ways of dealing with their health problems, such as calling physical and internet-based clinics. Also, they have connections with retired medical professionals and ask for help before using rural healthcare resources.” The participants openly talked about their difficulties in health issues and began to know and use new social and healthcare resources to manage their health problems. Participant 8 stated, “I was amazed because different participants have different resources to use for their health. I could acquire new ways of dealing with my symptoms.”

The process of acquiring new knowledge and skills regarding HSBs was based on continual reflection. Some participants continuously participated in health dialogues and reconsidered their HSBs several times. The process of reflection enhanced their learning about HSBs. Participant 1 stated, “Initially, I did not think I could reflect on my usual behaviors regarding HSBs. However, by participating in the health dialogue several times, I could consider my behaviors from various perspectives and was motivated to change my behaviors.” The new knowledge of HSBs included multiple social resources in rural communities. The participants were motivated to achieve effective health control in rural contexts. Participant 17 stated, “In reality, rural areas have various resources. I am satisfied and secure. I am motivated to use more health resources in rural communities for better health.”

### Familiarity with physicians improved motivation for safe and secure HSBs

Physicians’ participation in health dialogues changed rural citizens’ perceptions of their relationships with physicians. Physicians facilitated citizens’ discussions about their health conditions and listened to their symptoms at citizens’ pace. The situation was very different from that in medical institutions. Participant 17 stated, “In medical institutions, physicians are always busy and may not have time to listen to patients well. In this dialogue, they kindly listened to my symptoms and behaviors. I could say a lot about things during the dialogue.” Participant 2 stated, “Taking up with physicians here is beneficial for me. I knew the personality of each physician and changed their perception.” Through health dialogues with physicians, the participants could flexibly change their perception of physicians to a familiar existence.

The effective usage of relationships and collaborations help me act effectively to improve my health conditions. Rural areas have few medical professionals; therefore, rural citizens feel that physicians are far away from their lives and are inaccessible. Through dialogue, the participants realized that the physicians were familiar with them and that they could share their thoughts openly. Participant 22 stated, “I could say a lot of things to the physicians, and they replied to me kindly. I thought that physicians did not respond to me in-depth, and I just got a prescription in the medical institution.” Participant 9 stated, “Through this dialogue, I could change my image of physicians and say everything related to health. The image of physicians changed completely after communicating with them continuously.” The continual dialogue between citizens and physicians positively changed the physicians’ image and made them open-minded about medical issues.

Familiarization with physicians changed the image of rural medical institutions. Participants previously felt that rural medical institutions were busy and inaccessible to older people. Through dialogue with the physicians, they felt that rural medical institutions could be used effectively. Participant 12 stated, “For me, medical institutions were far from my life. These were inaccessible because of their estranged images. This dialogue was effective in changing the image of rural medical institutions.” The participants understood physicians’ working conditions and the differences in their attitudes between medical institutions and communities. They believed that they should adjust their perceptions and behaviors regarding their symptoms. Participant 6 stated, “Physicians are fundamentally busy in their working settings. However, they desired to listen to patients’ stories and approach their symptoms. I understand this point, so now I do not have difficulty accessing medical institutions.”

### Revising HSBs based on rural social resources

Health dialogues have emphasized the importance of connections in communities. The participants considered that rural contexts lack healthcare resources compared to urban areas. Mutual understanding and assistance among rural citizens are essential for effective HSBs. Participant 3 stated, “There are few hospitals, clinics, and pharmacies in rural areas. Public transportation systems are lacking. The sharing of knowledge of HSBs among rural citizens is fruitful. The connections among us should be driven for an effective control of health.” The connection among rural citizens was found to be a critical and valuable social resource.

Collaboration among rural residents must be improved to ensure effective connections. Improvement in collaboration through dialogue was considered adequate. Participant 11 stated, “Through this dialogue, I could know new ways of health control, but above all, the foundation is the relationship between us. I should frequently improve the collaboration with neighbors to share and effectively use healthcare resources in rural contexts.” For effective collaboration, social interactions need to increase. Owing to the COVID-19 pandemic, older people in rural contexts were reluctant to go out and meet others. The participants considered that their perception should be changed for better healthcare. Participant 8 said, “COVID-19 is dangerous, but I feel that going out and regaining social relationships with others in the community is better for improving health conditions.”

HSBs can be revised based on mutual understanding and effective collaboration among rural citizens. Social resources are lacking in rural areas. However, the participants realized that, based on their collaboration, the practical use of their knowledge and skills could improve their HSBs. Participant 21 stated, “The lack of social resources may be a drawback in rural areas, but I have social relationships among citizens. The effective usage of relationships and collaborations help me act effectively to improve my health conditions.”

## Discussion

This study clarified that rural health dialogues can help citizens share their experiences of HSBs and promote positive conversations by talking to others. Sharing their HSBs through health dialogues could facilitate rural citizens’ reflections on their own HSBs, realize their resources for HSBs, and consider revising their HSBs accordingly. In addition, dialogues with physicians in communities increased participants’ familiarity with physicians, which further improved their motivation for safe and secure HSBs.

Health dialogues are essential for driving health promotion in communities, increasing the opportunity to feel joy, and recognizing HSBs among older patients. COVID-19 restricted older patients’ mobility and interactions within communities, owing to their vulnerability and mortality from the infection [26–28]; they suffered from a lack of social interaction with relatives and friends in their communities [29]. The effects might be vital in rural contexts, where older patients live independently [29, 30]. Previous studies have shown that the COVID-19 pandemic has caused physical and psychological instability and increased frailty and depression in older patients [31, 32]. The lack of community dialogue also induced social isolation among older adults, further deteriorating their HSBs [33, 34]. As shown in this study, health dialogues facilitated by physicians can allow older people to see other community members and motivate them to consider improving their HSBs. As reported in previous articles, in the post-COVID-19 period, the sustainability of rural communities demands effective collaboration among citizens [35, 36]. The use of a joy-driven health dialogue, as suggested in this study, can be one of the solutions to increase social interactions and joy related to health in rural communities.

Reflection on one’s own HSBs could change the perception of others’ behaviors, from interest to intention, leading to better health. Regarding HSBs, the change in perception from interest to intention may be related to the QOL [9]. The current study showed that health dialogues can drive participants to reflect on and improve their intentions for HSBs in better ways. Thus, health dialogues can improve a participants’ QOL. The opportunities for dialogue regarding health conditions in communities may have decreased owing not only to COVID-19 but also to privacy issues [37, 38]. The latter in rural contexts may impinge on dialogues among older patients, thereby inhibiting collaborations among rural people [39]. In rural areas that lack social resources, collaborations among older adults are essential for the sustainability of healthcare [16]. An increase in opportunities for health dialogues can improve people’s health and should be driven by rural communities.

The use of social and healthcare resources should be considered to effectively revise HSBs in rural contexts. As shown in this study, the participants realized new social and healthcare resources through health dialogues. The realization of new social resources motivated the participants to revise their HSBs using the new resources. In rural contexts, healthcare resources are limited; therefore, older patients’ HSBs may also be limited [40]. Previous reports had shown that the limited resources impinge on older patients’ HSBs and their QOL [41, 42]. To improve HSBs, social and healthcare resources should be clarified in rural contexts and informed to older patients [3, 37]. As demonstrated in this study, rural contexts provide mutual help to communities, supporting people’s lives socially. In current medical care, there is an insufficient provision of information and clarification of social resources in rural contexts [11]. Local governments and communities should clarify these issues for effective HSBs and support for older patients [16].

Increasing familiarity with physicians in rural contexts can improve older patients’ motivation to secure HSBs. In rural contexts, older patients feel psychologically distanced from their physicians owing to their previous experiences [43, 44]. Due to the lack of medical resources and an imbalance between need and demand in rural contexts, physicians can perform their duties with hope and respect for patients’ opinions, without paternalism [45, 46]. Patients’ experiences of paternalism impinge on the image of medical professionals, making them appear arrogant and selfish [47]. Changing the image of medical professionals, especially physicians, is essential to improve healthcare in rural contexts. As shown in this study, continual health dialogues between physicians and citizens in rural contexts can positively modify the image of physicians. Changes in image can increase the familiarity of physicians and medicines in rural contexts [48, 49]. Mental accessibility to healthcare and HSBs influences the health conditions of older patients [9, 50]. This study demonstrated the possibility of improving rural health dialogues and effectively driving collaborations between citizens and physicians. Future studies should investigate changes in subjective and objective health outcomes through continual collaborations among communities.

The current study has several limitations. The first concern was the motivation of participants to learn about HSBs. The participants could have the motivation to learn HSBs. In this study, the researchers clarified the changing processes regarding the motivation of multiple participants through iterative data collection. Another limitation was transferability since this study was conducted only in rural Japanese communities. To improve the reliability of data, we used iterative data analysis and prolonged data collection. Future studies should investigate effective educational methods in other regions and international contexts, including this research theory. The first author coded the interview data, which may have affected the study’s credibility. To improve research quality, the second researcher reviewed the processes of coding, concepts, and themes through theoretical triangulation. Another limitation of this study is the focused exploration of dialogue, without a detailed investigation into other social determinants of health and their interaction with dialogue in influencing HSBs. Future studies could provide a more comprehensive analysis by considering the multifactorial nature of HSBs in rural settings.

## Conclusions

Rural health dialogues can help rural citizens share their experiences of HSBs and promote joyful conversations by talking to others. Sharing their HSBs through health dialogues could facilitate rural citizens’ reflection on their own HSBs, realize their resources for HSBs, and consider revising their HSBs based on rural social resources. In addition, dialogues with physicians in communities increased participants’ familiarity with physicians, which improved their motivation for safe and secure HSBs. The possibility of improving rural health dialogues and effectively driving collaborations between citizens and physicians should be investigated in the future.

## Data Availability

The datasets used and/or analyzed in the current study are available from the corresponding author upon reasonable request.

## List of abbreviations

HSBs: help-seeking behaviors
QOL: quality of life
